# Extraperitoneal laparoscopy for para-aortic lymphadenectomy in endometrial carcinoma staging: an approach with higher efficiency

**DOI:** 10.1186/s12957-021-02416-x

**Published:** 2021-11-07

**Authors:** Wei Zhang, Lingfang Xia, Xiaotian Han, Xingzhu Ju, Xiaohua Wu, Xiaojun Chen

**Affiliations:** Department of Gynecologic Oncology, Fudan University Shanghai Cancer Center, Shanghai, China

**Keywords:** Endometrial carcinoma, Extraperitoneal, Transperitoneal, Laparotomy, Minimally invasive, Lymphadenectomy

## Abstract

**Background:**

Removing more inframesenteric nodes is not only significantly increases the likelihood of finding metastasis for endometrial cancer, but also can add survival advantage. As most patients diagnosed with endometrial cancer are overweight or obesity, a high efficiency approach is important. Aim of this study was to compare the surgical outcomes of extraperitoneal laparoscopic, transperitoneal laparoscopic, and laparotomic para-aortic lymphadenectomy in endometrial carcinoma staging.

**Methods:**

We retrospectively reviewed data of all patients diagnosed with primary endometrial carcinoma who were treated at the Department of Gynecologic Oncology, Fudan University Shanghai Cancer Center from 1 January 2017 to 31 December 2019. The numbers of para-aortic lymph nodes, surgical time, complications, blood loss and hospital stay were compared. The patients’ medical records and pathological reports were carefully reviewed. Statistical significance was defined as *p* < 0.05.

**Results:**

We retrospectively compared patients who underwent extraperitoneal laparoscopy (Group E, *n* = 20), transperitoneal laparoscopy (group T, *n* = 21), and laparotomy (group L, *n* = 135). The median number of para-aortic lymph nodes was significantly higher in group E than in groups T and L (9.5, 5, and 6, respectively; *p* = 0.004 and 0.0004, respectively). All patients in group E underwent successfully dissection to the renal vessel level. The median operation time was significantly shorter in group L than in groups T and E (94, 174, and 233 min, respectively; *p* < 0.0001). The median estimated blood loss volume was higher in group L than in groups T and E (200, 100, and 142.5 ml, respectively; all comparisons *p* < 0.001), and the length of hospital stay was significantly longer in group L than in Groups T and E (6, 5, and 6 days, respectively; all comparisons *p* < 0.001).

**Conclusion:**

The extraperitoneal laparoscopic approach for staging endometrial carcinoma harvested higher numbers of para-aortic lymph nodes which could be considered for endometrial carcinoma staging, especially for para-aortic lymph node harvest.

**Supplementary Information:**

The online version contains supplementary material available at 10.1186/s12957-021-02416-x.

## Background

Transperitoneal laparoscopic lymphadenectomy was first reported in the early 1990s for surgical staging [1]. As confirmed by the Gynecologic Oncology Group (GOG), the transperitoneal laparoscopic approach is a satisfactory method for surgical staging of endometrial carcinoma [2]. The extraperitoneal approach was first reported in 1995 and was primarily used to evaluate para-aortic metastases in patients with cervical cancer [1, 3, 4]; more attention has been given to staging of patients with endometrial cancer via this approach in recent years [5, 6]. Although laparoscopic surgery is the preferred approach for endometrial cancer, with less blood loss and a shorter length of hospital stay [7], minimally invasive approaches were reportedly used by only 60% of the gynecological oncologists [8].

Computed tomography, magnetic resonance imaging (MRI) and positron-emission tomography (PET-CT) were used for evaluation of lymph node status. The positive predictive value (PPV) and negative predictive value (NPV) of MRI in detecting lymph node metastasis have been reported to be 47.1 to 78%, and 57 to 94.4%, respectively [9]. For PET-CT was with 50% PPV and 97.4% NPV [10]. For women with a completely negative PET scan, 12% had disease spread to nodes along the aorta [11]. According to these results, surgical evaluation of lymph node status is necessary. However, 50% of the surgeons routinely stopped at the inferior mesenteric artery if lymphadenectomy was needed, and only 11% reported routinely dissecting nodes to the level of the renal vessels (RVs) [8]. Considering that 77% of patients with endometrial carcinoma with positive para-aortic lymph nodes have metastases above the inferior mesenteric artery [7], and because as many as 10% of patients with clinically early endometrial carcinoma have infrarenal node involvement [12]. Turan et al. [12] reported tumor grade, histologic type, and myometrial invasion cannot be used as markers to decide on supramesenteric lymphadenectomy in endometrial cancer; therefore, the upper limit for para-aortic lymphadenectomy must be the left RV. Removing more inframesenteric nodes significantly increases the likelihood of finding cancer metastasis for endometrial cancer [13], and dissecting the infrarenal nodes during staging can add a 10% survival advantage [14].

The left aortic nodes contain 63% of all aortic nodes, which makes the left-sided extraperitoneal technique much easier and suitable for lymph node dissection in patients with endometrial cancer [15, 16]. With its lower risk of intestinal and urinary injury and adhesions, the extraperitoneal technique is considered superior to the transperitoneal approach in both obese and non-obese patients [17]. One study showed that in non-obese patients, the transperitoneal approach was associated with a higher number of harvested lymph nodes [17].

To date, despite the studies published comparing the different lymphadenectomy approaches [7, 18, 19], only a few studies focused on staging of endometrial carcinoma. Our aim was to compare the surgical outcomes including numbers of para-aortic lymph nodes, surgical time, intra- and post-operative complications, blood loss, and hospital stay of systemic staging of endometrial carcinoma via extraperitoneal, transperitoneal laparoscopic, and laparotomic approaches.

## Methods

### Patients and inclusion criteria

We retrospectively collected and reviewed data for all patients diagnosed with primary endometrial carcinoma who were treated at the Department of Gynecologic Oncology, Fudan University Shanghai Cancer Center, from 1 January 2017 to 31 December 2019. This study was conducted until 1 April, 2020. We included data for patients who underwent systemic staging, including para-aortic lymphadenectomy, and excluded patients who did not undergo para-aortic lymphadenectomy. Patients with a synchronous primary ovarian tumor were also excluded. Clinical information, physical examination notes, operation records, and pathology records were carefully reviewed. Tumor stage was classified according to the 2009 International Federation of Gynecology and Obstetrics (FIGO) staging system. Approval to review the patients’ medical records and pathological reports was obtained from the institutional review board of Fudan University Shanghai Cancer Center, Shanghai, China (050432-4-1911D). Written informed consent was obtained from all patients. All patients’ data were anonymized to avoid patient information identification during or after data collection. Baseline characteristics of these patients in each group including age, BMI, and stage were compared to ensure their balance.

### Surgical procedure

Systemic staging involved hysterectomy, salpingo-oophorectomy, and pelvic and para-aortic lymphadenectomy. Omentectomy was performed in patients with grade 3 primary endometrioid uterine carcinoma and in those with non-endometrioid carcinoma. Radical hysterectomy was performed when the cervix was involved, which was confirmed using magnetic resonance imaging or biopsy. Because the renal vein was routinely used as the upper margin for lymphadenectomy by our team, all node dissections were attempted to the level of the RVs regardless of the approach. For laparoscopic procedures, the aortic and pelvic nodes were put into a plastic specimen bag and was extracted either virginally or through a 15-mm port [1]. Vaginal manipulator was used when hysterectomy was performed.

Surgery was performed in two steps, for transperitoneal and laparotomy group, first hysterectomy and pelvic lymphadenectomy, and then para-aortic lymph node dissection. And for extraperitoneal group, para-aortic lymph node dissection was performed first, and then hysterectomy and pelvic lymphadenectomy.

The procedure for extraperitoneal laparoscopic para-aortic lymphadenectomy was performed as previously reported, with simple modifications [20, 21]. Briefly, a 10-mm incision was made in the umbilicus for constant visual control during development of the extraperitoneal space. Then, to develop the extraperitoneal space, a 15-mm incision was placed 2 cm superomedially to the anterosuperior iliac spine. Once the peritoneal layer was visualized, the surgeon introduced one finger into the incision and performed blunt dissection to separate the peritoneal layer from the abdominal wall, under constant visual control via the umbilical port [20]. Once the extraperitoneal space was formed, a 15-mm trocar was inserted and gas was insufflated into the extraperitoneal space at a pressure of 12 to14 mmH g[20, 21]. A 5-mm and 10-mm trocar was placed at the midaxillary line, and another 5-mm trocar was placed if needed; four to five trocars were placed in total. Posteriorly, peritoneal marsupialization was performed with a 2- to 3-cm incision to prevent formation of lymph cysts was performed to prevent the formation of lymph cysts. Lymphadenectomy were performed before hysterectomy in extraperitoneal laparoscopy.

The operating time was recorded from skin incision to closure, and included all procedures performed in each group. We also recorded the blood loss volumes for the three procedures. Intra-operative and postoperative complications were studied. Postoperative complications were defined as any complications occurring within 90 days after surgery.

All the surgeons in our department were well trained and skilled in both laparotomy and minimally invasive procedures, and all procedures were done by the same experienced gynecologic oncologists. Histopathologic diagnosis was made and reviewed by 2 experienced pathologists.

### Statistical analysis

The patients’ age and body mass index (BMI) were compared using one-way ANOVA post hoc test, surgical stage, number of harvested para-aortic lymph nodes, surgical time, estimated blood loss, and hospital stay were compared using the Mann-Whitney *U* test in each paired group. Fisher’s exact test was used to compare categorical variables between groups. A *p* value of < 0.05 was considered statistically significant. SPSS and MedCalc version 11.1.2.0 (MedCalc Software, Ostend, Belgium) was used for all statistical analysis.

## Results

**Fig. 1:**
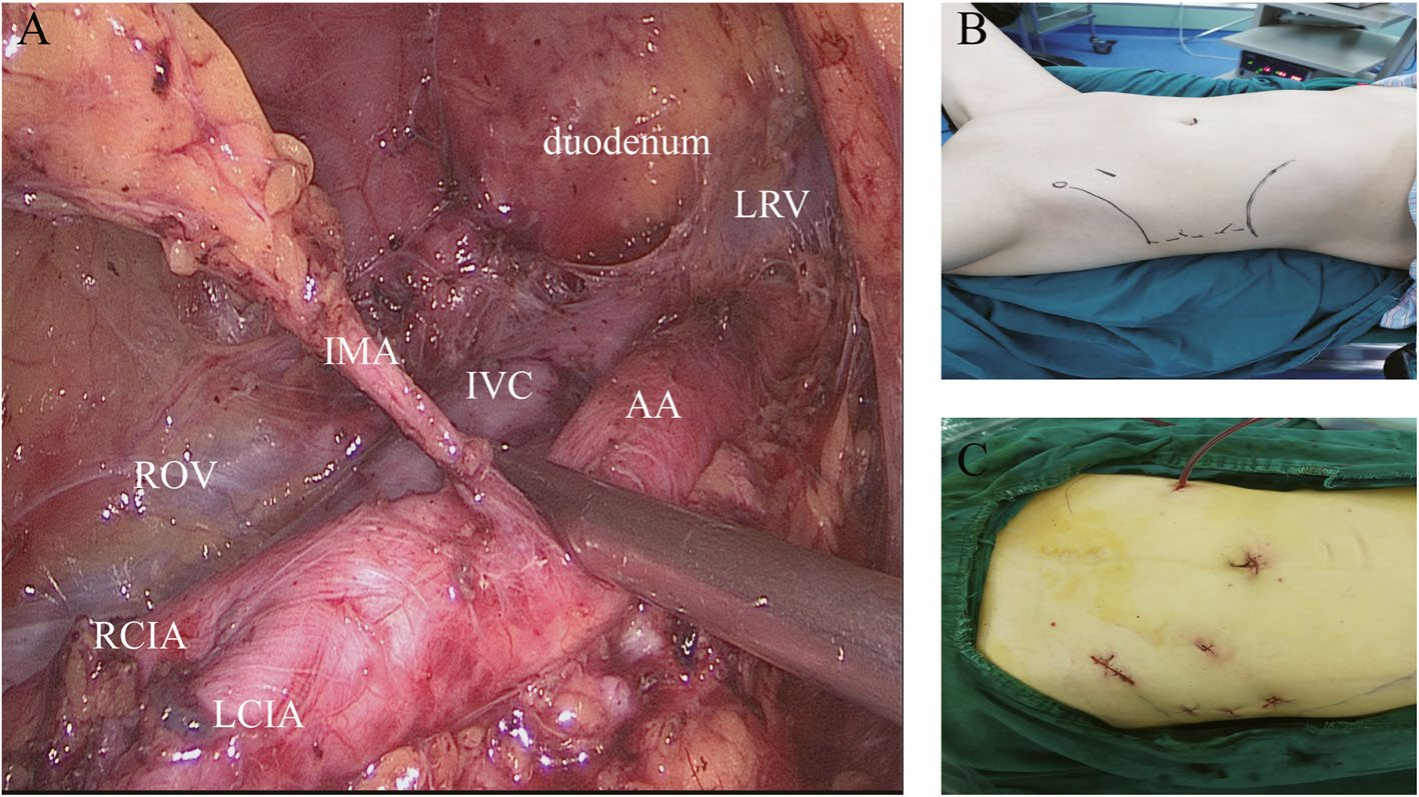
**A** View from the left side during the extraperitoneal approach, with the duodenum and left renal vein (LRV) as the roof of the dissection cavity. The inferior mesenteric artery (IMA) emanates from the abdominal aorta (AA), which accompanied the inferior vena cava (IVC). **B** Incision position for extraperitoneal laparoscopy

We reviewed the data of 176 patients who underwent primary surgical staging treatment for endometrial cancer. The patients were divided into a transperitoneal laparoscopic para-aortic lymphadenectomy group (group T, *n* = 21), laparotomic para-aortic lymphadenectomy group (group L, *n* = 135), and extraperitoneal laparoscopic para-aortic lymphadenectomy group (group E, *n* = 20). View from the left side during the extraperitoneal approach and position of each trocar placed is presented in Fig. 1.

The patients’ characteristics are summarized in Table 1. The patients’ median age at surgery was similar in Group T, Group L, and Group E (52, 54, and 55 years, respectively; all comparisons, *p* > 0.05). The median BMI was also similar in group T, L, and E (24.35, 24.22, and 25.45 kgm^2^, respectively; all comparisons, *p* > 0.05). The most common histological type was endometrioid adenocarcinoma in all groups, and grade 1 was the most common tumor grade in all three groups. The FIGO stage was also comparable in the three groups (stages I and II vs stages III and IV, all comparisons, *p* > 0.05), and stage IA was the most common stage.

**Table 1:** Patients characteristics

Patient characteristics	group T	groupL	group E	***P*** value
	*n* = 21	*n* = 135	*n* = 20	
**Age at surgery,yrs**				
median(range)	52(39-70)	54(32-75)	55 (41-69)	All *p* > 0.05
**BMI,kg/m2 (median,range)**	24.35(16.9-32.51)	24.22(16.41-50.78)	25.45(21.19-32.51)	
<18 ,n(%)	1(4.76%)	1(0.74%)	0(0%)	
18-25,n(%)	13(61.9%)	76(56.3%)	8(40%)	
25-30,n(%)	6(28.57%)	45(33.33%)	10(50%)	
30-35,n(%)	1(4.76%)	11(8.15%)	2(10%)	
>35,n(%)	0(0%)	2(1.48%)	0(0%)	
**Surgical stage**				
IA	14(66.67%)	90(66.67%)	12(60%)	stage I and II vs stage III and IV all *p* > 0.05
IB	3(14.29%)	14(10.37%)	5(25%)	
II	2(9.52%)	10(7.4%)	1(5%)	
IIIA	2(9.52)	3(2.22%)	0(0%)	
IIIB	0(0%)	1(0.74%)	0(0%)	
IIIC1	0(0%)	11(8.15%)	1(5%)	
IIIC2	0(0%)	4(2.96%)	1(5%)	
IVA	0(0%)	0(0%)	0(0%)	
IVB	0(0%)	(2.96%)	0(0%)	
**Histologic grade (G)**				
G1	9(42.86%)	55(40.74%)	9(45%)	distribution of tumor grades, All *p* > 0.05
G2	9(42.86%)	44(32.59%)	6(30%)	
G3	1(4.76%)	19(9.63%)	3(15%)	
UPSC	0(0%)	8(5.93%)	1(5%)	
CCC	0(0%)	7(5.19%)	0(0%)	histopathological types, *p* > 0.05
MMMT	0(0%)	0(0%)	0(0%)	
Gx	2(9.52%)	0(0%)	0(0%)	
Mixed epithelial carcinoma	0(0%)	1(0.74%)	0(0%)	
Dedifferentiated carcinoma	0(0%)	10.74%)	1(5%)	

Footnote: *BMI* body max index, *UPSC* uterineserous carcinoma, *CCC* clear cell carcinoma, *MMMT* malignant mullerian mixed tumor

The numbers of para-aortic nodes harvested via the different procedures are presented in Table 2. The median number of para-aortic lymph nodes was significantly higher in group E than in groups T and group L (9.5, 5, and 6, respectively; *p* = 0.004 and *p* = 0.0004, respectively). There was no significant difference between group T and L (*p* = 0.581). Patients in group L had more positive aortic nodes than patients in groups T and E (30, 0, and 1 respectively). All patients in group E underwent successful dissections to the RV level, and 71.43% and 94.07% of the patients in groups T and L , respectively. The rate of dissection to the RV level was significantly higher in groups L and E than in group T (*p* = 0.0043 and *p* = 0.02, respectively), but the difference between groups L and E was not statistically significant (*p* = 0.597). After limiting the count of removed lymph nodes to the group of patients who have dissected the para-aortic region up to the RV level, median number of harvested para-aortic node is 7, 6, and 9.5 in groups T, L, and E, respectively. Group E harvest more node than group L (*p* = 0.0005), but not significantly different with group T (*p* = 0.082).

**Table 2:** Patients characteristics of para-aortic node dissection via different procedure

	groupT	groupL	groupE	
	*n* = 21	*n* = 135	*n* = 20	
**No. of para-aortic lymph nodes**				group T vs group L, *P* = 0.581,group T vs group E, *P* = 0.004,group L vs group E, *P* = 0.0004
median(range)	5(1-21)	6(1-19)	9.5 (5-29)	
**positive aortic nodes**	0(0%)	30(22.22%)	1(5%)	
**cases with positive aortic nodes, no.(%)**	0(0%)	6(4.44%)	1(5%)	
**cases with dissection to RV level**	15(71.43%)	127(94.07%)	20(100%)	group T vs group L, *P* = 0.0043,group T vs group E, *P* = 0.02,group L vs group E, *P* = 0.597

Perioperative and postoperative details are shown in Table 3. The median operation time was significantly shorter in group L than in groups T and E (96 min, 174 min, and 233 min, respectively; *p* < 0.0001). The difference between groups T and E was also significant (*p* = 0.001).

**Table 3:** Perioperative and postoperative characteristics

	group T	group L	group E	***P*** Value
	*n* = 21	*n* = 135	*n* = 20	
Surgical time,min*				*group T vs group L, *P* < 0.0001 *group T vs group E, *P* = 0.001 *group L vs group E, *P* <0.0001
median(range)	174(93-275)	96(51-212)	233.5(145-309)	
**intraoperative complications,no(%)**				
urinary injury	1(4.76%)	2(1.48%)	0	
**estimated blood loss,ml**^**#**^ **median(range)**	100(50-200)	200(50-1000)	142.5(50-300)	^#^group T vs group L, *P* < 0.0001 ^#^group T vs group E, *P* >0.05 ^#^group L vs group E, *P* = 0.0086
**Blood transfusion (unit)**				
mean(range)	0(0%)	0.17(0-4)	0	
**Surgical procedure**				
hysterectomy	19(90.48%)	117(86.67%)	19(95%)	
radical hysterectomy	2(9.52%)	18(13.33%)	0(0%)	
omentectomy	0(0%)	23(17.04%)	3(15%)	
appendectomy	0(0%)	5(3.7%)	1(5%)	
CRS	0(0%)	1(0.74%)	0(0%)	
**hospital stay,days**^**※**^				
median(range)	5 (3-11)	6(4-21)	6(5-7)	^**※**^group T vs group L, *P* < 0.0001 ^**※**^group T vs group E, *P* = 0.0781 ^**※**^group L vs group E, *P* < 0.0001
**postoperative complications**				
paralutic ileus	0(0%)	1(0.74%)	0(0%)	
asymptomatic lymphocele	0(0%)	10(7.4%)	0(0%)	
lymphedema	0(0%)	1(0.74%)	0(0%)	

The estimated blood loss volumes were higher in group L than in groups T and E (200 ml, 100 ml, and 142.5 ml, respectively; all comparisons, *p* < 0.001), but difference between groups T and group E was not statistically significant (*p* > 0.05).

The median length of hospital stay was significantly longer in group L than in groups T and E (6, 5, and 6 respectively; all comparisons, *p* < 0.001). The length of hospital stay was similar in groups T and E (*p* = 0.078).

Urinary injury was the most common intraoperative complication in groups T and L, whereas no patients sustained a urinary injury in group E. Patients in Group L developed the most postoperative complications, namely paralytic ileus (0.74%), asymptomatic lymphocele formation (7.4%), and lymphedema (0.74%). Only one patient was converted from group E to group T because of peritoneal leakage, and this patient was analyzed as part of group T.

## Discussion

Thorough staging of endometrial carcinoma not only results in accurate documentation of disease spread, but is also a therapeutic intervention [13]. Minimally invasive techniques have recently been proven safe alternatives for surgical staging, with less morbidity and quick recovery. The extraperitoneal laparoscopic lymphadenectomy approach provides equally favorable outcomes, especially regarding reaching the supramesenteric lymph nodes [5]. The upper limit of para-aortic lymphadenectomy must be the left renal vein because many studies have shown that clinically early endometrial carcinoma may have positive infrarenal nodes [12, 22, 23]. For this reason, our team routinely used the renal vein as the upper margin for lymphadenectomy.

The key finding of our study is that after comparing the transperitoneal, laparotomic, and extraperitoneal approaches, the extraperitoneal laparoscopic approach was most satisfactory for staging endometrial carcinoma, especially regarding para-aortic lymphadenectomy.

This study also showed that extraperitoneal laparoscopic para-aortic lymphadenectomy harvested higher numbers of para-aortic nodes than the other two approaches. This might be because of the left-side extraperitoneal technique provides easier access to the left aortic nodes, which contains 63% of all aortic nodes [15, 16].The extraperitoneal approach allows improved access to the left aortic lymph nodes, especially to the challenging supramesenteric nodal group without bowel interfering. A previous study suggested harvesting 5.3 to 21 aortic nodes. We harvested a median of 9.5 para-aortic lymph nodes in group E, 5 in group T, and 6 in group L. Pakish et al. [24] reported a mean of 5 nodes harvested using the transperitoneal approach and 10 nodes using the extraperitoneal approach. The authors also indicated that extraperitoneal laparoscopy harvested significantly higher numbers of para-aortic lymph nodes than the transperitoneal approach. Dowdy et al. [20] found that the total number of harvested para-aortic lymph nodes was not significantly different between extraperitoneal laparoscopy and laparotomy. Although there was no difference in the rate of dissection to the RV level between group L and group E, these two approaches were significantly different in number of nodes harvest. Therefore, we believe that difference of removed lymph nodes numbers not only related to the level of lymph node dissection, but also related to the technique.

Removing more inframesenteric nodes significantly increases the likelihood of finding cancer, as does increasing the numbers of dissected infrarenal nodes [13]. Although we harvested more para-aortic lymph nodes in group E, the percentage of positive nodes was higher in group L. This result might be explained by the larger portion of grade 3 tumors and non-endometrial carcinomas in group L, including papillary serous carcinoma, clear cell carcinoma, and malignant mixed mesodermal tumors, which are considered high-risk and more likely to metastasize to lymph nodes. Recently, increasingly more studies have been showing that lymphadenectomy does not benefit patients with grades 1 and 2 endometrioid lesions with myometrial invasion of ≤ 50% and a primary diameter of ≤ 2 cm [25]. Thus, performing more limited dissection of nodes or sentinel nodes, or even harvesting nodes might be safe in low-risk patients [26–28]. SLN (sentinel lymph node) mapping in early-staged EC has been demonstrated to be safe and accurate [28]. Low rate of lymph node metastasis in our results also support this, and limited node dissection might be adopted in our further work.

Time-consuming was a major disadvantage of the extraperitoneal approach in our study. Examination of the intraoperative outcomes revealed no significant difference in the total operative time between the groups in a previous study [5], similar to our results. The operative time in group E was 30 min longer than that in Group T and 130 min longer than that in group L. As reported previously, the extraperitoneal laparoscopic approach was associated with significantly shorter operative times for lymphadenectomy, whereas the total operative times were not different between the two groups [5]. The longer time might be related to the time required to prepare the retroperitoneal space. Previous studies showed that the total operative time with the extraperitoneal approach ranged from 200 to 339.5 min [1, 18, 24], and our results were similar. Surgery in both group T and group E required more time than with laparotomy, which might reflect surgical skill.

The estimated blood loss volumes were lowest in group T, and the length of hospital stay was shortest in group E. Group L had significantly higher blood loss volumes and significantly longer hospital stays. These findings are consistent with the Gynecologic Oncology Group’s recommendation to perform minimally invasive approaches for endometrial cancer because of less blood loss and shorter length of hospital stay [7].

The most frequently reported postoperative complications after lymph node dissection are lymphocele and lymphedema [17], and paralytic ileus is also not uncommon. In group E, we routinely performed peritoneal marsupialization to prevent the formation of lymph cysts. We found that group E had few complications and low failure rates; only one patient required conversion to transperitoneal laparoscopy, similar to previous studies [20, 29].

Our study has several limitations; the first limitation is the retrospective nature of the clinical data and the single-center design. Second, the sample size in group E was small, and the small number of patients with a BMI of > 35 kg/m^2^ limited the BMI-correlated subgroup analysis. Therefore, our results are limited to patients with a BMI of < 35 kg/m^2^. Additionally, because we began performing extraperitoneal laparoscopy (group E) only within 3 years of this study, we did not analyze overall survival in each group. As most of the patients in our study were stage 1A, whether these results are applicable to patients with higher stages of disease still need further study.

## Conclusion

Our study showed that endometrial carcinoma staging via the extraperitoneal laparoscopic approach resulted in higher numbers of dissected para-aortic lymph nodes, longer surgical time, fewer intraoperative and postoperative complications, and shorter hospital stays than the laparotomy approach. The extraperitoneal laparoscopic approach should be considered for surgical staging of endometrial carcinoma.

## Supplementary Information

**Additional file 1.**

## Data Availability

The dataset supporting the conclusions of this article is included within the article and its additional file.
